# Initiation-specific alleles of the Cdc45 helicase-activating protein

**DOI:** 10.1371/journal.pone.0214426

**Published:** 2019-03-26

**Authors:** Ramon Y. Rios-Morales, Sze Ham Chan, Stephen P. Bell

**Affiliations:** Howard Hughes Medical Institute, Department of Biology, Massachusetts Institute of Technology, Cambridge, MA, United States of America; University of Minnesota Twin Cities, UNITED STATES

## Abstract

The committed step in DNA replication initiation is the activation of the Mcm2-7 replicative DNA helicase. Two activators, Cdc45 and GINS, associate with Mcm2-7 at origins of replication to form the CMG complex, which is the active eukaryotic replicative helicase. These activators function during both replication initiation and elongation, however, it remains unclear whether Cdc45 performs the same function(s) during both events. Here, we describe the genetic and biochemical characterization of seven Cdc45 mutations. Three of these mutations are temperature-sensitive lethal mutations in *CDC45*. Intriguingly, these mutants are defective for DNA replication initiation but not elongation. Consistent with an initiation defect, all three temperature-sensitive mutants are defective for CMG formation. Two of the lethal mutants are located within the RecJ-like domain of Cdc45 confirming the importance of this region for Cdc45 function. The remaining two lethal mutations localize to an intrinsically disordered region (IDR) of Cdc45 that is found in all eukaryotes. Despite the lethality of these IDR substitution mutants, Cdc45 lacking the IDR retains full function. Together, our data provide insights into the functional importance of Cdc45 domains and suggest that the requirements for Cdc45 function during DNA replication initiation are distinct from those involved in replication elongation.

## Introduction

Eukaryotic DNA replication requires the sequential loading and activation of the heterohexameric replicative Mcm2-7 DNA helicase at origins of replication. During G1, Mcm2-7 hexamers are loaded onto origins of replication as head-to-head double hexamers encircling double-stranded DNA (dsDNA) [1–6]. The initially loaded Mcm2-7 complexes remain inactive until the cell enters S-phase. During S-phase, Dbf4-dependent kinase (DDK) and S-phase cyclin-dependent kinase (S-CDK) promote the association of two helicase activators, Cdc45 and the GINS complex, with loaded Mcm2-7 complexes [7]. Briefly, DDK phosphorylation of Mcm4 and Mcm6 promotes the association of Cdc45 and Sld3/7 [8–11]. S-CDK phosphorylates Sld2 and Sld3 to facilitate recruitment of a complex between Sld2, Dpb11, Polymerase ε (Pol ε) and GINS to Mcm2-7, Cdc45 and Sld3/7 [12–14]. These associations drive formation of the active replicative helicase, the Cdc45-Mcm2-7-GINS (CMG) complex [15]. Final activation of the CMG complex requires binding of Mcm10 and results in the generation of regions of single-stranded DNA (ssDNA) sufficient for the recruitment of the rest of the DNA replication machinery [10, 16–20].

Helicase activation requires dramatic remodeling of the loaded Mcm2-7 double hexamer. Although the initially loaded Mcm2-7 complexes are dimeric and encircle dsDNA [1–2, 21–22], in the context of the CMG, Mcm2-7 functions as a single hexamer that encircles ssDNA [23–25]. Thus, during Mcm2-7 activation, the double-hexamer interface must be disrupted, the DNA within Mcm2-7 must be unwound and the lagging-strand DNA template ejected from each Mcm2-7 complex. Structural studies have captured the Mcm2-7 complex at different stages of helicase loading and also in the context of the CMG [22–23, 26–30]. These structures have identified elements of the double-hexamer structure likely to contribute to initial DNA unwinding and reveal that Cdc45 and GINS bridge the entry site for dsDNA into the Mcm2-7. Recent biochemical studies suggest that formation of the CMG is sufficient to drive initial DNA unwinding and transition to a form of the complex encircling ssDNA [18]. Nevertheless, the conformational changes required for initial DNA melting, transition from the inactive Mcm2-7 double hexamer to the single-Mcm2-7-containing CMG complex, and the role of Cdc45 during these events remain elusive.

Cdc45 is a critical helicase activator that is required for both DNA replication initiation and elongation [10, 31–34]. Cdc45 binds to the N-terminal domain of Mcm2-7 at the interface between the Mcm2 and Mcm5 subunits [23, 26, 29], which opens to allow dsDNA entry to the Mcm2-7 central channel [3, 35–36]. Together with GINS, Cdc45 is proposed to stabilize a closed-ring state of the N-terminal domains of Mcm2-7 while the C-terminal ATPase ring switches between a closed and cracked-ring conformation [29, 32], although in the presence of DNA the CMG has only been observed in a closed form [26]. In addition, Cdc45 interacts with Pol ε in the context of the CMGE [37–38], suggesting that Cdc45 also facilitates association between the active helicase and the leading-strand DNA polymerase.

Although the effects of Cdc45 loss during DNA replication have been studied, how Cdc45 functions during helicase activation and elongation remains to be fully understood. In particular, little is known about the Cdc45 domains that mediate these processes. *In vitro* studies demonstrate that Cdc45 and GINS association stimulates Mcm2-7 ATPase and helicase activity suggesting one mechanism for helicase activation [15]. In addition, Cdc45 and GINS binding drives initial DNA unwinding at the origin but Mcm10 is required for more extensive DNA unwinding [18]. In contrast, in vivo studies suggest that initial DNA unwinding is dependent on Mcm10 activity [16, 19–20]. It is possible that prior to Mcm10 function, Cdc45 stimulates CMG ATPase activity but the CMG is maintained in a conformation not competent for extended DNA unwinding. Importantly, Cdc45 function is also essential for DNA elongation as Cdc45 degradation at replication forks halts DNA replication elongation rendering cells unable to finish S-phase [34].

In this study, we designed and tested Cdc45 mutations to investigate the mechanism of Cdc45 function. Using both genetic and biochemical approaches, we characterized mutations that separate Cdc45 function during DNA replication initiation from its role during DNA elongation. We also provide evidence that the regions of Cdc45 related to RecJ and specific residues in an intrinsically-disordered region are essential for Cdc45 function. Our findings suggest that the requirements for Cdc45 function during DNA replication initiation are distinct from those involved in DNA elongation and further our understanding of the functional domains of Cdc45.

## Materials and methods

### Yeast strains and *CDC45* mutant construction

All nineteen *CDC45* mutants were constructed by site directed PCR mutagenesis and confirmed by sequencing. Expression vectors for Cdc45 protein purification were constructed by site-directed PCR mutagenesis of pMM33. All *Saccharomyces cerevisiae* strains were congenic with W303 (*ade2-1 trp1-1 leu2-3*,*112 his3-11*,*15 ura3-1 can1-100*). The strains and plasmids used in this manuscript are summarized in *S1* and *S2 Tables.* The function of mutant *CDC45* genes was tested using a Cdc45 ‘swapper’ strain (yBC093) in which the chromosomal copy of *CDC45* was deleted and a wild-type copy of *CDC45* maintained in plasmid harboring a *URA3* marker. Each mutant gene was integrated into the *LEU2* locus of the swapper strain and tested for viability on media containing 5-Fluorootic acid (5-FOA) that selected against the presence of the *CDC45* plasmid. Integration of each *cdc45* mutant was confirmed by PCR. Mutant gene expression was under control of the endogenous *CDC45* promoter.

### Flow cytometry

Cell cultures were inoculated in YPD medium and grown overnight at 25°C. Cultures were then diluted to OD_600_ of 0.05 and grown for 3h at 25°C in YPD medium. Where indicated cells were arrested with α-factor (1 μg/mL) for 3h at 25°C. Cells were released from the α-factor arrest by washing twice with fresh YPD medium including 50 μg/mL pronase (YPD^pronase^; EMD Millipore). For experiments that shift to the non-permissive temperature during the α-factor arrest, cells were shifted to 37°C 1h after the initial α-factor arrest at 25°C. Subsequent α-factor release, which occurred after a total of three hours arrest, was performed by washing with and transferring into YPD^pronase^ prewarmed to 37°C. For HU-arrest experiments, cells were initially arrested in α-factor then released from the α-factor arrest by washing with YPD^pronase^ and then resuspended in 25°C YPD^pronase^ with 200 mM hydroxyurea (Acros Organics) at 25°C for 30 min. Cells were released from hydroxyurea by washing three times with prewarmed to 37°C YPD medium and then resuspended in 37°C YPD medium. Samples (1 mL) were centrifuged, the supernatant removed and the pellet resuspended in 1 mL of 70% ethanol. Cells were centrifuged a second time and resuspended in 500 μL of 50 mM Sodium Citrate with 0.02 mg/mL RNAseA and incubate for 2 h at 50°C. Proteinase K (0.08 mg/ml) was then added to each sample followed by incubation for an additional 2 h at 50°C. The resulting samples were sonicated (Branson [Danbury, CT USA], Model 250 with microprobe, five 2 second pulses at 20% amplitude), and the DNA labeled with Sytox green (ThermoFisher) and analyzed using flow cytometry (CytoFlex, Beckman Coulter).

### Protein purification

ORC, Cdc6, and Mcm2-7/Cdt1 were purified as previously described [39]. DDK, Dpb11, Pol ε, S-CDK, Mcm10 and Pol α-primase were purified as previously described [17]. RPA was purified from yeast as described in [40]. Sld2, Sld3-Sld7, GINS, and wild-type or mutant Cdc45 were purified as follows.

All yeast protein expression strains were grown in selective medium before being inoculated into 8 L (12 L for Sld2) of YEP + 2% glycerol at 30°C. Cells were grown to an OD_600_ of ~1 before addition of galactose (2% final concentration). After 4–6 h of growth, cells were harvested and washed with 250 mL of 50 mM HEPES (7.6), 1 M Sorbitol and 1 mM PMSF. The cells were then resuspended in approximately one-half the packed cell volume with the indicated lysis buffer containing Roche protease inhibitor cocktail. The resuspended cells were frozen drop-wise in liquid nitrogen. The frozen cells were lysed using a freezer/mill (SPEX SamplePrep). The lysed cell powder was transferred to ultracentrifugation tubes and thawed on ice. The lysate was cleared by centrifugation at 160,000 g for 1 h. All purification steps were done at 4°C.

Cleared cell lysates from cells overexpressing Flag-tagged proteins were incubated with the indicated amount of packed anti-Flag M2 affinity gel (Sigma) for 2 h at 4°C. Bound proteins were washed with the indicated buffers and eluted with the indicated buffer with 0.2 mg/mL 3xFlag peptide (MDYKDHDGDYKDHDIDYKDDDDK; Swanson Biotechnology Center, Koch Institute). The first eluate was collected by flowing one column volume (CV) of elution buffer over resin. After a 30 min incubation, one CV of elution buffer was added to the column and the elution collected. This process was repeated four times.

#### Cdc45

Cdc45-3xFlag was overexpressed from yMM16. Purification of Cdc45 was based on a previously published protocol [17] with modifications. Cells expressing the Cdc45 temperature-sensitive mutants were grown at 25°C to avoid protein instability induced by growth at higher temperatures. Cells were resuspended in buffer H (50 mM HEPES-KOH at pH 7.6, 5 mM MgOAc, 10% glycerol) with 0.5 M potassium glutamate (KGlut), 1 M sorbitol, 3 mM ATP, Roche protease inhibitor cocktail and 1 mM PMSF. After lysis, the lysate was incubated with 1.5 mL of anti-Flag M2 affinity gel equilibrated with buffer H + 0.5 M KGlut, 2 mM ATP. The resin was washed with 20 CV of buffer H + 0.5 M KGlut, 2 mM ATP, 1mM PMSF followed by 10 CV of 20 mM potassium phosphate buffer (pH 7.4), 0.15 M KOAc, and 10% glycerol (Buffer C). Cdc45 was eluted in buffer C with 3xFlag peptide. Fractions were pooled and loaded onto a 1.5 mL hydroxyapatite column previously equilibrated with buffer C. The column was washed with a buffer containing 80 mM potassium phosphate (pH 7.4), 0.15 M KOAc, and 10% glycerol and eluted with a buffer containing 0.3 M potassium phosphate (pH 7.4), 0.15 M KOAc, and 10% glycerol. Cdc45 fractions were pooled and dialyzed against buffer H with 0.3 M KGlut.

#### GINS

GINS was purified from a yeast strain overexpressing Sld5, Psf1, Psf3, and Psf2-3C-6xHis-Flag (ySK136). Cells were resuspended in buffer H with 0.5 M KCl, 1M sorbitol, 0.02% NP-40, 2 mM ATP, Roche protease inhibitor cocktail and 1 mM PMSF. After lysis, the cleared lysate was diluted to 0.3 M KCl with buffer H with Roche protease inhibitor cocktail. The lysate was then incubated with 1.5 mL of anti-Flag M2 affinity gel equilibrated with buffer S (buffer H with 0.3 M KCl and 0.02% NP-40). The resin was washed with 20 CV of buffer S followed by 10 CV of buffer H with 0.02% NP-40 and 0.1 M KCl. GINS was eluted in the final wash buffer with 3xFlag peptide. GINS-containing fractions were pooled and the Flag tag on Psf2 was removed with an overnight incubation (16 h) with HRV 3C protease (45 nM) at 4°C. The resulting protein was flowed over a one mL Complete His-tag column to remove undigested GINS and His-tagged HRV 3C protease before applying the flow-through to a one mL HiTrap Q HP column (GE healthcare). GINS was eluted with a 20 CV gradient of 0.1–1 M KCl in buffer H with 0.02% NP-40. Peak fractions were dialyzed against buffer H with 0.3 M potassium KOAc and 0.02% NP-40.

#### Sld3/7

Sld3-Sld7 was purified from a strain overexpressing Sld3(105–668)-3xFlag and Sld7-VSV-G (ySK123). The deletion of residues 1–104 of Sld3 removes a potential destruction box. Cells were resuspended in buffer H with 0.8 M KCl, 1M sorbitol, 0.02% NP-40, 2mM ATP, Roche protease inhibitor cocktail and 1mM PMSF. After cell lysis, the cleared lysate was diluted with buffer H and protease inhibitors to a conductivity equal to buffer H with 0.3 M KCl. The diluted lysate was incubated with 1.5 mL of anti-Flag M2 affinity resin equilibrated with buffer H with 0.3 M KCl. The resin was washed with 30 CV of buffer H with 0.3 M KCl and eluted in buffer H with 0.3M KCl and 3xFlag peptide. Sld3/Sld7-containing fractions were diluted to 0.2 M KCl with buffer H and applied to a one mL HiTrap SP HP column (GE Healthcare). The column was washed with buffer H with 0.3 M KCl, 0.02% NP-40 and eluted with buffer H with 0.66 M KCl, 0.02% NP-40. Sld3/7 fractions were pooled and dialyzed against H + 0.3 M KCl, 0.02% NP-40.

#### Sld2

Sld2 was purified from a yeast strain overexpressing 3xFlag-3C-6xGly-Sld2 (ySK127). Cells were resuspended in buffer H with 0.8 M KCl, 1M sorbitol, 0.02% NP-40, 2 mM ATP, Roche protease inhibitor cocktail and 1mM PMSF. After cell lysis, the cleared lysate was dialyzed overnight (16 h) in buffer H with 0.3 M KCl, 3 mM ATP and 1mM PMSF and cleared by centrifugation at 25,000 x g for 20 min. The lysate was incubated with 1.5 mL of anti-Flag M2 affinity gel equilibrated with buffer H with 0.3M KCl, 1mM ATP. The resin was washed with 30 CV of the same and eluted in buffer H with 0.3 M KCl + 3xFlag peptide. Sld2-containing fractions were diluted with buffer H to a conductivity equivalent to Buffer H with 0.2 M KCl and applied to a one mL HiTrap SP HP column. Sld2 was eluted with a 15 CV gradient from 0.2 to 1 M KCl in buffer H, 0.02% NP-40, and 1 mM ATP.

### Reconstituted DNA replication assay

The DNA plasmid template p*ARS1*-Nco (7.6 kB) was linearized, biotinylated and coupled to streptavidin-coated magnetic beads as described previously [10]. All incubations were performed in a Thermomixer (Eppendorf) at 1250 rpm and 25°C. A DynaMag-2 magnet (ThermoFisher Scientific) was used to collect DNA beads and remove the supernatant after each step. Mcm2-7 loading was performed by incubating with 45 nM ORC, 45 nM Cdc6 and 100 nM of Mcm2-7/Cdt1 with 120 fmol of bead-attached p*ARS1*-Nco in 25mM HEPES (pH 7.6), 10 mM MgOAc, 2 mM DTT, 300 mM KGlut, 20 mM creatine phosphate, 5 mM ATP, 5% glycerol, 0.02% NP-40 and 0.2 μg of creatine kinase in a 10 μL reaction for 25 min. Beads were collected, the supernatant removed and DDK phosphorylation was performed in a 10 μL reaction containing 50 mM HEPES (pH 7.6), 3.5 mM MgOAc, 1 mM DTT, 225 mM KGlut, 1 mM ATP, 10% glycerol, 0.02% NP-40 and 130 nM DDK for 25 min. DNA replication was initiated by adding the following to the DDK reaction: 30 nM S-CDK, 62 nM Sld2, 40 nM Dpb11, 25 nM Pol α-primase, 30 nM Pol ε, 50 nM RPA, 200 nM GINS, 2.5 nM Mcm10, 50 nM Sld3/7, 130 nM Cdc45, 0.2 mM rNTPs, 0.04 mM dNTPs and 10 μCi [α-^32^P] dCTP in a 20 μL containing 40 mM HEPES (pH 7.6), 10 mM MgOAc, 2 mM DTT, 250 mM KGlut, 20 mM creatine phosphate, 3 mM ATP, 8% glycerol, 0.02% NP-40, 400 ug/mL BSA and 0.2 μg of creatine kinase. DNA replication reactions were allowed to proceed for 1h. The magnetic beads were captured, washed with buffer H with 300 mM KGlut and 0.02%NP-40. DNA was released from beads by boiling in SDS-PAGE sample buffer and products were separated in a 0.8% alkaline agarose gel and imaged using a phosphor screen. For immunoblot analyses, proteins were released from the bead-associated DNA by incubating with 5U of DNase (Worthington) in 15 μL of buffer H with 200 mM NaCl, 2 mM CaCl_2_, 0.01% NP-40 for 30 min at 25°C before immunoblotting. The following antibodies were used for immunoblotting: α-Mcm2-7 (UM174), α-Cdc45 (HM135), α-GINS (HM128), and α-Pol ε (HM7602). Immunoblots were quantified using ImageJ followed by a one-way ANOVA multiple comparison analysis using the Prism software (GraphPad).

### CMG formation assay

The DNA plasmid template pUC19-*ARS1* (3.8kB) was biotinylated and coupled to streptavidin-coated magnetic beads as described previously [17]. These reactions were performed as described for the reconstituted DNA replication assay with the following changes: 60 fmol of DNA was incubated with 45 nM ORC, 45 nM Cdc6 and 100 nM of Mcm2-7/Cdt1 in 25 mM HEPES (pH7.6), 10 mM MgOAc, 2 mM DTT, 225 mM KGlut, 20 mM creatine phosphate, 5 mM ATP, 5% glycerol, 0.02% NP-40 and 0.2 μg of creatine kinase. After 25 min, 130 nM DDK was added directly to the reaction and incubated for an additional 25 min. After DDK phosphorylation, beads were collected and the supernatant was removed. CMG formation was initiated by adding 30 nM S-CDK, 62 nM Sld2, 40 nM Dpb11, 30 nM Pol ε, 200 nM GINS, 2.5 nM Mcm10, 50 nM Sld3/7 and 50 nM Cdc45 in a 10 μL reaction containing 25 mM HEPES (pH 7.6), 10 mM MgOAc, 2 mM DTT, 250 mM KGlut, 5 mM ATP, 8% glycerol, 0.02% NP-40 and 400 ug/mL BSA. CMG assembly reactions were incubated for 30 min at 25°C in a Thermomixer at 1250 RPM. Magnetic DNA beads were captured and washed with buffer H with 300 mM KCl and 0.02% NP-40. Proteins were released from the DNA by incubating with 5U of DNase (Worthington) in 15 μL of buffer H with 200 mM NaCl, 2 mM CaCl_2_, 0.01% NP-40 for 30 min at 25°C before immunoblotting. Immunoblots were quantified using ImageJ (N.I.H.) followed by one-way ANOVA multiple comparison analysis using the Prism software (GraphPad).

## Results

### Characterization of *CDC45* site-directed mutants

**Fig 1:**
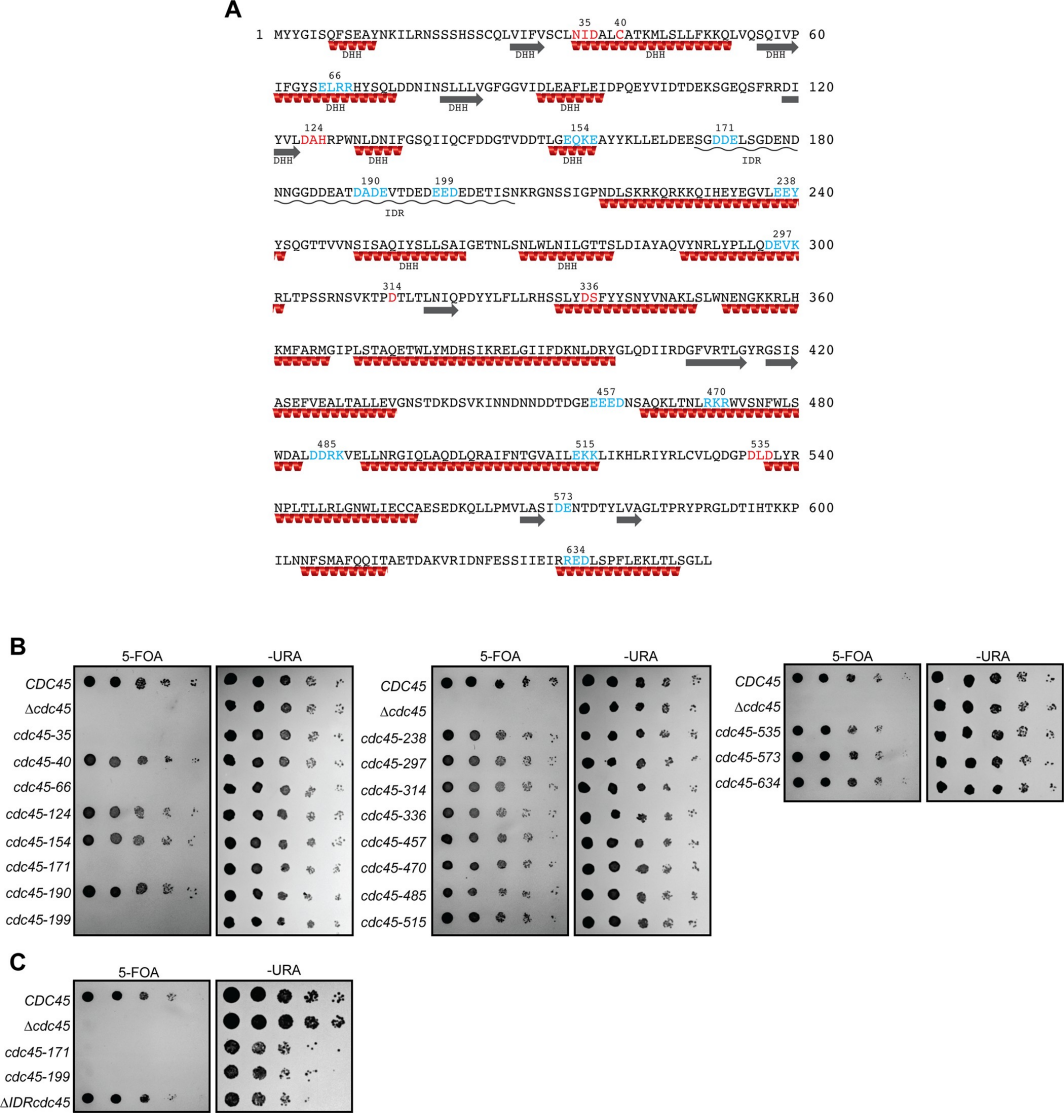
*In vivo* analysis of *CDC45* mutants. (A) Site-directed mutants of *Saccharomyces cerevisiae* Cdc45 are shown in either red (mutation of conserved sequeces) or blue (mutation of clusters of charged amino acids). Mutants are numbered based on the position of the first mutated amino acid. All targeted residues were mutagenized to alanine. Red spirals and gray arrows denote α-helices and β-strands, respectively (adapted from Yuan, Z., et al., 2016). The α-helices and β-strands belonging to the DDH domain from RecJ were adapted from Simon, A.C., et al (2016). The intrinsic disorder region (IDR) of Cdc45 is underlined with a black wavy line. (B) Four out of nineteen *cdc45* mutants are lethal. Five-fold serial dilutions of cells containing the indicated *CDC45* allele before (-URA) or after (5-FOA) loss of the plasmid with WT *CDC45* were grown on indicated plates for 3d at 25°C.(C) The CDC45 IDR is dispensable for cell viability. Mutants in the Cdc45 IDR, *cdc45-171* and *cdc45-199*, are lethal. Cells were analyzed as described in (B).

To better understand the role of Cdc45 during DNA replication, we designed nineteen *CDC45* clustered point mutations (Fig 1A). Sites were selected for mutagenesis using either conservation from yeast to humans (S1 Fig) or alanine-scanning mutagenesis of clusters of charged amino acids [41]. For the latter method, we mutated regions with three charged amino acids in a four-residue window with the goals of targeting surface-exposed residues and avoiding null mutants that disrupt the hydrophobic core of a protein. The resulting mutant *cdc45* genes were tested for their ability to complement a *CDC45* deletion. Four of the nineteen mutants were unable to support cell viability (Fig 1B). None of the viable mutants were sensitive to hydroxyurea (HU) or methyl methanesulfonate (MMS, S2 Fig).

Two of the lethal mutations are located in the conserved N-terminal region of Cdc45 that is homologous to the bacterial exonuclease RecJ (S1 and S3 Figs) [42–43]. The *cdc45-35* mutation alters one of the four regions related to the RecJ DHH active site motifs (Asn-Ile-Asp motif) and *cdc45-66* is located in an α-helix within a region related to the RecJ DHH domain. The amino acids mutated in *cdc45-35* and *cdc45-66* do not form intermolecular interactions with the Mcm2-7 complex or GINS in the context of the CMG [29]. Consistent with the hypothesis that these mutations cause defects in Cdc45 protein folding, we were unable to purify soluble versions of these mutant proteins.

Given the impact of mutants in the DHH domain on Cdc45 function, we asked if restoring the DHH catalytic triad impacted *CDC45* function. The DHH (Asp-His-His) catalytic triad motif in RecJ is mutated to DAH in *S*. *cerevisiae* and other Cdc45 proteins. Consistent with this difference, no exonuclease activity has been reported for Cdc45 [44]. However, the similar structures of RecJ and the Cdc45 N-terminus and previously detected weak interactions between hCdc45 and DNA [43] suggested that restoring the DHH catalytic motif in Cdc45 could restore exonuclease activity leading to DNA damage and lethality. To test this possibility, we restored the DHH motif in *ScCDC45*, however, this allele did not show any defects in cell viability or growth (S4 Fig). Thus, despite similarities between the RecJ and Cdc45 N-terminal domains, it is unlikely that the Cdc45 RecJ domain is able to form an active nuclease even if the DHH motif is restored.

The remaining two lethal mutants, *cdc45-171* and *cdc45-199*, are located in a region of Cdc45 predicted to be disordered (S1 and S3 Figs) [45–46]. Consistent with this prediction, this region of Cdc45 is not resolved in recent structural studies of the CMG complex [23, 29]. In *S*. *cerevisiae*, this region is located just C-terminal of the RecJ region of homology and spans amino acids 169–229. This region contains a nuclear-localization signal (amino acids 210–229) but this motif is not altered by either mutation. Although the primary sequence in this region is poorly conserved, computational models identify a similarly-located intrinsically-disordered region (IDR) in Cdc45 from all eukaryotes analyzed (S5 Fig). To test if this disordered region is required for Cdc45 function, we deleted amino acids 169–209 (*cdc45ΔIDR*, the NLS at 210–229 was retained) and tested this mutation for complementation. In contrast to the lethal phenotype of the point mutations in the IDR, *cdc45ΔIDR* complemented a *CDC45* deletion (Fig 1B). Recent studies identified two phosphorylation sites in the IDR region of Cdc45 that mediate the recruitment of Rad53 to replication forks [47]. Interestingly, Cdc45 mutants that disrupt this interaction were not sensitive to HU or MMS despite being defective for Rad53 phosphorylation. Consistent with these results, the *cdc45ΔIDR* was not sensitive to HU (S6 Fig).

### Cdc45 temperature-sensitive mutants are selectively defective for DNA replication initiation

**Fig 2:**
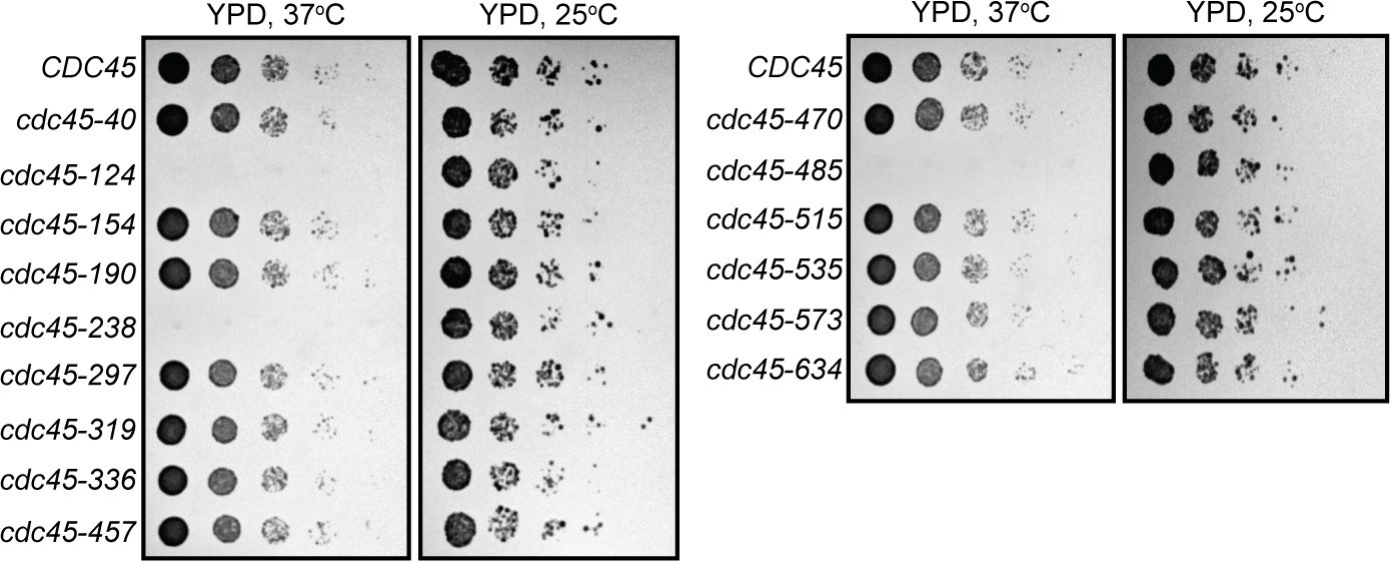
Three *CDC45* mutants are lethal when grown at 37°C. Five-fold serial dilutions of cells containing the indicated *CDC45* mutation as the only copy of *CDC45* were grown on YPD plates for 2d at 37°C or 3d at 25°C.

We next asked if any of the *CDC45* mutations that were viable at 25°C were defective for growth at elevated temperatures. Serial dilution of the viable *cdc45* mutants grown at 37°C revealed three *CDC45* alleles that exhibited temperature-sensitive (ts) growth defects (Fig 2). Based on structural studies, these *cdc45*^*ts*^ mutants do not cluster to a particular region in Cdc45 but are rather located in three different regions of the protein.

**Fig 3:**
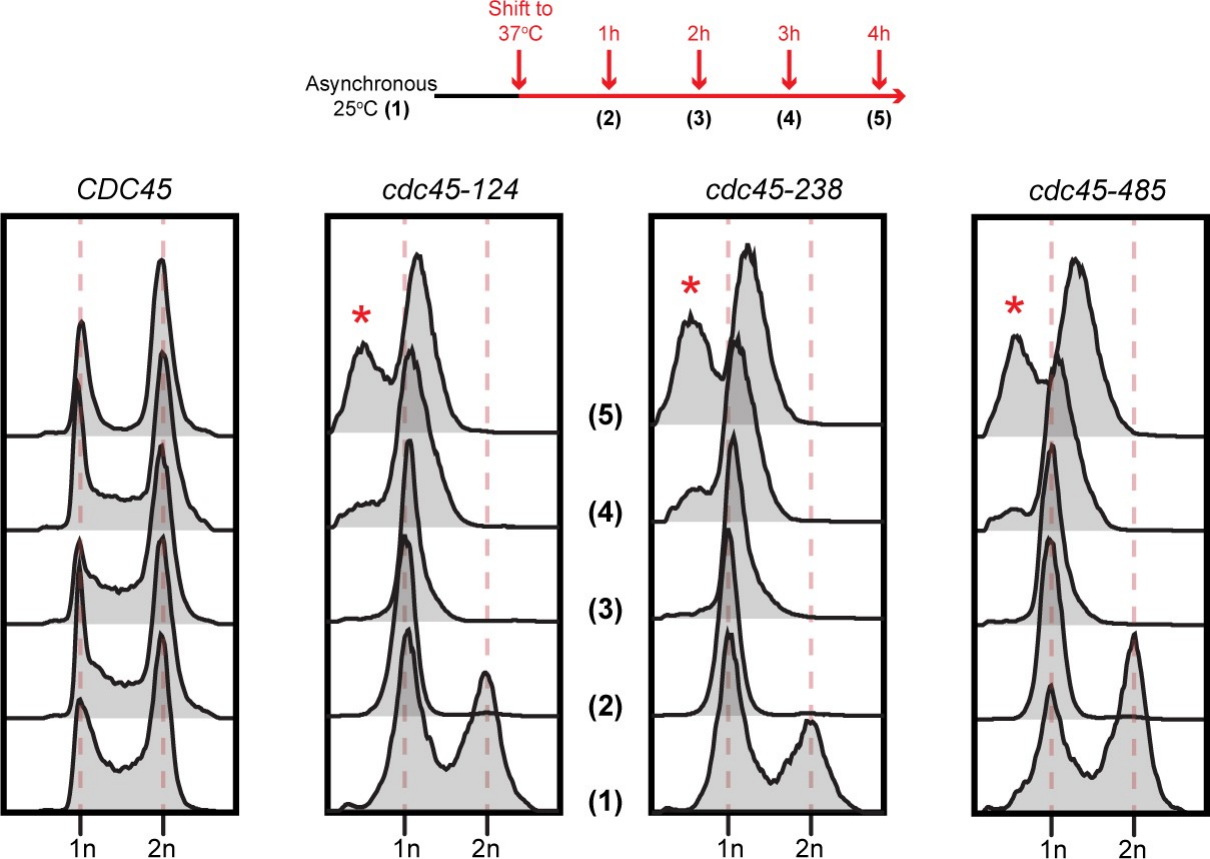
Temperature-sensitive alleles of *cdc45* accumulate cells with G1 DNA content at the restrictive temperature. *(Top)* Experimental scheme; asynchronous cultures of the indicated *cdc45* mutants were grown at 25°C in YPD and shifted to 37°C for 4h. *(Bottom)* Samples collected at indicated time points were analyzed using flow cytometry to assess their DNA content. The red asterisk denotes cells with less than G1 DNA content due to reductional anaphase.

To explore the consequences of inactivation of these temperature-sensitive mutants, we asked if they were defective for DNA replication initiation, elongation or both. Previous studies of degron mutations in Cdc45 found that Cdc45 is required for both replication initiation and elongation [34]. As a first test, we shifted cells containing *CDC45* or one of the three *cdc45*^*ts*^ mutations growing asynchronously at the permissive temperature to the restrictive temperature and monitored cellular DNA content using flow cytometry. Cells containing *CDC45* continued to divide and were distributed between cells with unreplicated, replicating or replicated DNA (Fig 3). In contrast, each of the ts-mutant-containing cells rapidly accumulated with G1 DNA. Because cells with defects in replication elongation would be expected to arrest throughout S-phase, the accumulation of cells with G1 DNA content suggested that these *cdc45* alleles were defective in DNA replication initiation but competent for elongation. In addition, all three *cdc45*^*ts*^ accumulated cells with G1 DNA content within the first cell cycle after shifting to the non-permissive temperature. Interestingly, at later time points cells with the *cdc45*^*ts*^ alleles accumulated cells with sub-G1 DNA content. This phenotype is characteristic of cells that arrest with fully unreplicated chromosomes and undergo reductional anaphase, a process during which unreplicated chromosomes are randomly segregated into the daughter cells [34, 48]. Exhibition of reductional anaphase strongly suggests these mutants completely prevent entry into S phase at the non-permissive temperature.

**Fig 4:**
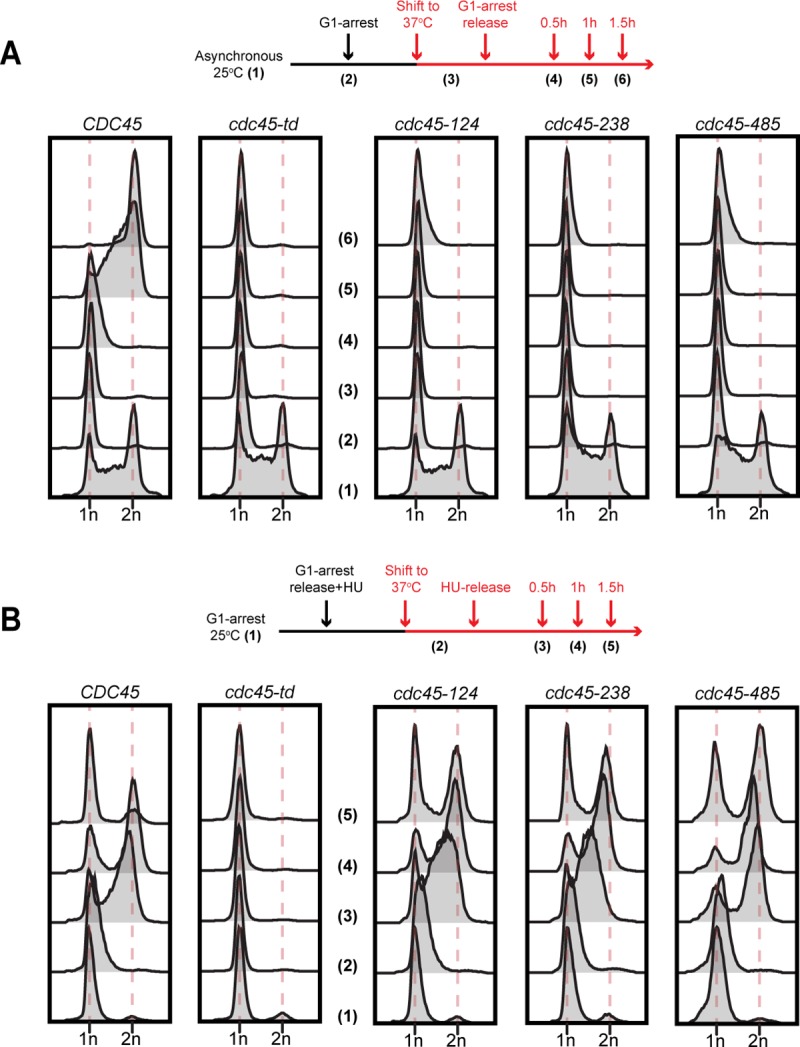
Temperature-sensitive alleles of *cdc45* are defective for DNA replication initiation. (A) *(Top)* Experimental scheme; asynchronous cells were arrested in G1 using α-factor at 25°C. After one hour of arrest, cells were shifted to 37°C and released from the arrest after two additional hours. *(Bottom)* Images of samples taken at the indicated time points and analyzed for DNA content using flow cytometry. (B) *(Top)* Experimental scheme; cells were arrested in G1 at 25°C for 3 hours and released from G1 arrest into YPD medium containing 200mM Hydroxyurea (HU) for 30min. After 30min of HU treatment, cells were shifted to 37°C for an additional 1h followed by release into 37°C YPD medium lacking HU. *(Bottom)* Images of samples harvested at indicated times and analyzed for DNA content using flow cytometry.

To test for an initiation defect more directly, we asked if the *cdc45-ts* mutant cells enter S-phase after inactivation during G1. Wild-type *CDC45* cells enter S-phase and finished DNA replication after α-factor arrest and release at the restrictive temperature (Fig 4A). In contrast, cells containing a temperature-sensitive degron allele of *CDC45* (*cdc45-td*) [34] failed to enter S-phase and maintained G1 DNA content after the α-factor release. Similar to the *cdc45-td* mutant, each of the *cdc45*^*ts*^ mutants showed strong defects in S-phase entry after α-factor arrest and release at the restrictive temperature, maintaining G1 DNA content 1.5 hours after G1 release (Fig 4A). These results suggest that all three *cdc45*^*ts*^ mutants are defective for DNA replication initiation.

To further address the hypothesis that the three *cdc45*^*ts*^ mutants are selectively defective for DNA replication initiation, we asked whether these mutants are competent for DNA replication elongation. In these experiments, we arrested cells at the permissive temperature in early S-phase using hydroxyurea (HU) and then raised them to the non-permissive temperature. We then monitored progress through S-phase after HU release at the non-permissive temperature. Wild-type *CDC45* cells resumed DNA replication, finished S-phase and underwent mitosis after HU release at the restrictive temperature. As previously shown, under the same conditions *cdc45-td* mutant cells failed to resume DNA replication and remained in S-phase [34]. Unlike inactivation during G1, inactivation of the three *cdc45*^*ts*^ mutants during S-phase did not inhibit completion of S-phase (Fig 4B). Flow cytometry revealed that *cdc45-124*, *cdc45-238* and *cdc45-485* mutants resumed DNA elongation and finished S-phase in a time frame similar to wild-type cells. Taken together, these findings indicate that *cdc45-124*, *cdc45-238* and *cdc45-485* mutants are defective in DNA replication initiation but competent for replication elongation at the non-permissive temperature.

### Temperature-sensitive Cdc45 proteins are defective for DNA replication and CMG stability *in vitro*

To assess the underlying defects of the Cdc45-124, Cdc45-238 and Cdc45-485 mutant proteins, we purified and tested the function of these proteins using a reconstituted DNA replication assay. Briefly, purified helicase-loading, helicase-activation, and replication-elongation factors were sequentially incubated with origin-containing DNA attached to beads [17, 40]. Protein complex formation was assayed by monitoring DNA-retained proteins. DNA synthesis was monitored through incorporation of radiolabeled dCTP followed by separation of the replication products on a denaturing agarose gel. Although it is possible that the mutant defects would be more obvious at 37°C, these assays were performed at 25°C as even with wild-type Cdc45 the assay was compromised at higher temperatures. Note that these assays were performed without lagging-strand enzymes (e.g. DNA Pol δ) and therefore lead to primarily leading-strand products.

**Fig 5:**
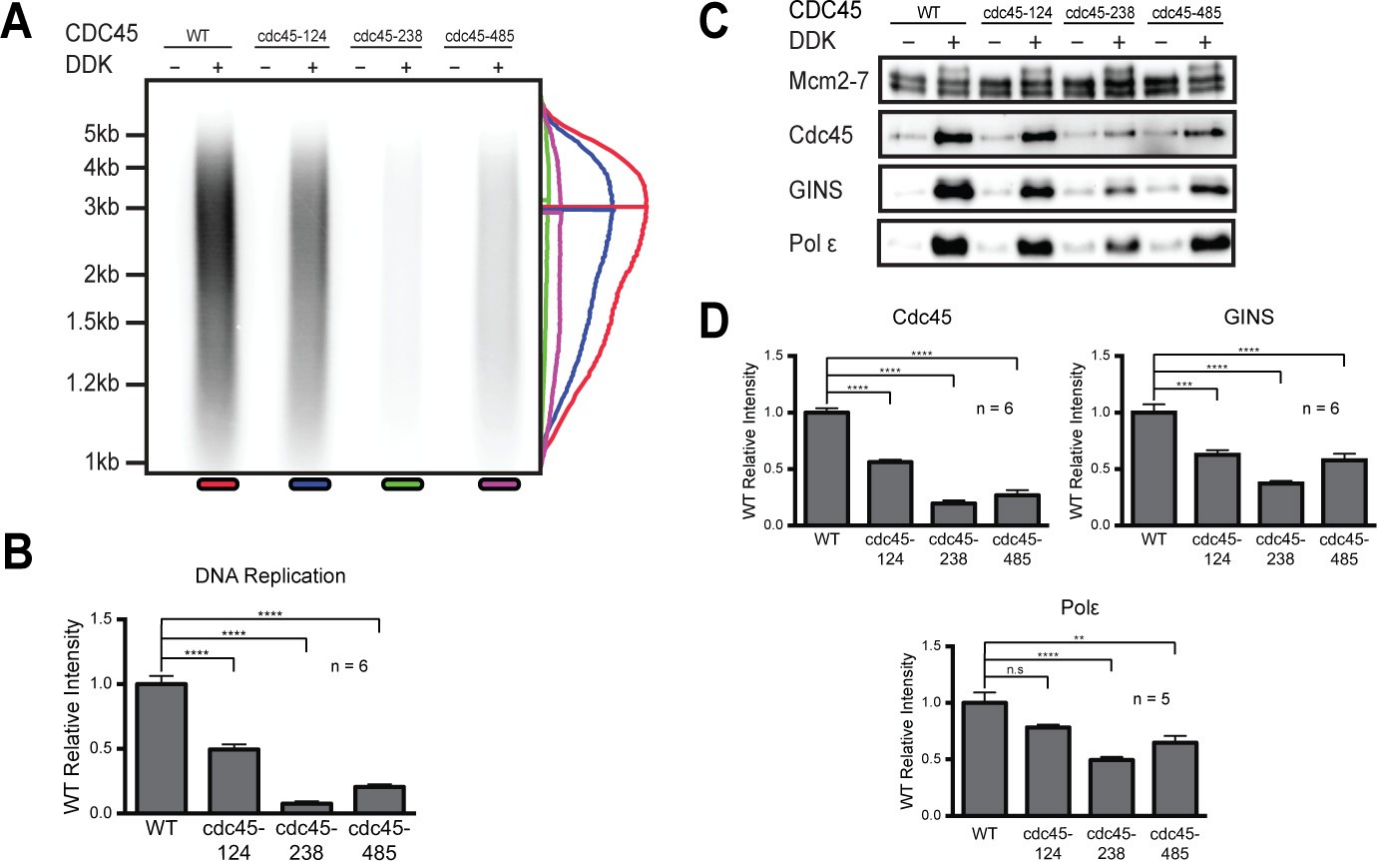
Cdc45-ts mutants are defective for DNA replication. (A) DNA replication products produced with the indicated Cdc45 proteins were separated on a 0.8% alkaline agarose gel and imaged using a phosphoimager. Relative intensities of +DDK lanes were quantified and plotted using ImageJ (Cdc45 = red, Cdc45-124 = blue, Cdc45-238 = green, Cdc45-485 = purple). Horizontal lines indicate the most highly represented product length for each Cdc45 protein tested. (B) Relative levels of DNA replication for the indicated Cdc45 proteins from six experimental replicates of replication assays performed with the indicated Cdc45 mutant proteins were quantified and plotted. (C) Proteins associated with the DNA at the end of the replication reaction. Bead-associated proteins were washed with Buffer H + 0.3M K-Glut 0.02%NP-40, released with DNase, and detected by immunoblot. (D) Relative association of Cdc45, Mcm2-7, GINS and Pol ε with origin DNA after replication. Six (Cdc45 and GINS association) and five (Pol ε association) experimental replicates were quantified and plotted. For both (B) and (D), error bars represent standard error from the mean. Asterisks indicate the following p-values: p≤0.01(**), p≤0.001(***), p≤0.0001(****), not significant (n.s., p≥0.05).

As expected, wild-type Cdc45 showed robust DDK-dependent DNA replication (Fig 5A, Lane 1 and 2). In contrast, all three Cdc45 mutants were defective for DNA replication, but the extent of the defect varied between the mutants (Fig 5). The strongest defects were observed for Cdc45-238 and Cdc45-485 (8% and 20% of WT replication, respectively; Fig 5A and 5B). In contrast, Cdc45-124 had a weaker impact (~50% relative to WT Cdc45). Importantly, although the amount of products varied between WT and mutant Cdc45 proteins, the distribution of the lengths of the DNA replication products was similar for the wild-type and mutant Cdc45 proteins (Fig 5A, see plot on right of image of replication products). This observation is consistent with these mutants being defective in DNA replication initiation (resulting in less products) but not elongation (resulting in similar length products). It is possible that defects in DNA elongation by these mutants, such as a slower rate of synthesis, could be masked by our end-point analysis of DNA replication products. However, defects in DNA elongation has been shown in similar end-point analyses making this possibility unlikely [17].

To further investigate the molecular defects of these mutants, we analyzed the replication proteins that remained associated with the DNA at the end of the replication assay (Fig 5C). Consistent with the DNA replication experiments (Fig 5A), wild-type Cdc45 showed robust DNA association with the DNA at the end of the replication reaction (Fig 5C, lane 1–2). In addition, we also observed strong association of both GINS and Pol ε. In contrast, all three Cdc45 mutants showed reduced DNA association of these proteins (Fig 5C, lanes 3–8). Similar to the extent of replication defects observed, the amount of Cdc45-124 associated with origin-DNA was approximately half of wild-type levels (Fig 5D). Cdc45-238 and Cdc45-485 showed stronger defects with 20% and 27% of wild-type Cdc45 association, respectively (Fig 5D). Consistent with a requirement for Cdc45 association for their association, GINS and Pol ε DNA association were reduced to a similar extent as Cdc45 association for each mutant (Fig 5C and 5D). Taken together, these results are consistent with the Cdc45 mutants being defective for initial association with Mcm2-7. Previous studies showed that Cdc45 association with the Mcm2-7 complex is required for stable GINS recruitment and helicase activity [10, 15]. Thus, it is also possible that mutant Cdc45-ts proteins are competent for initial Mcm2-7 recruitment but defective for stable GINS association (i.e. CMG formation) and the lack of this interaction destabilizes association of Cdc45 with the DNA template.

### Cdc45 temperature-sensitive mutants are defective for CMG formation

**Fig 6:**
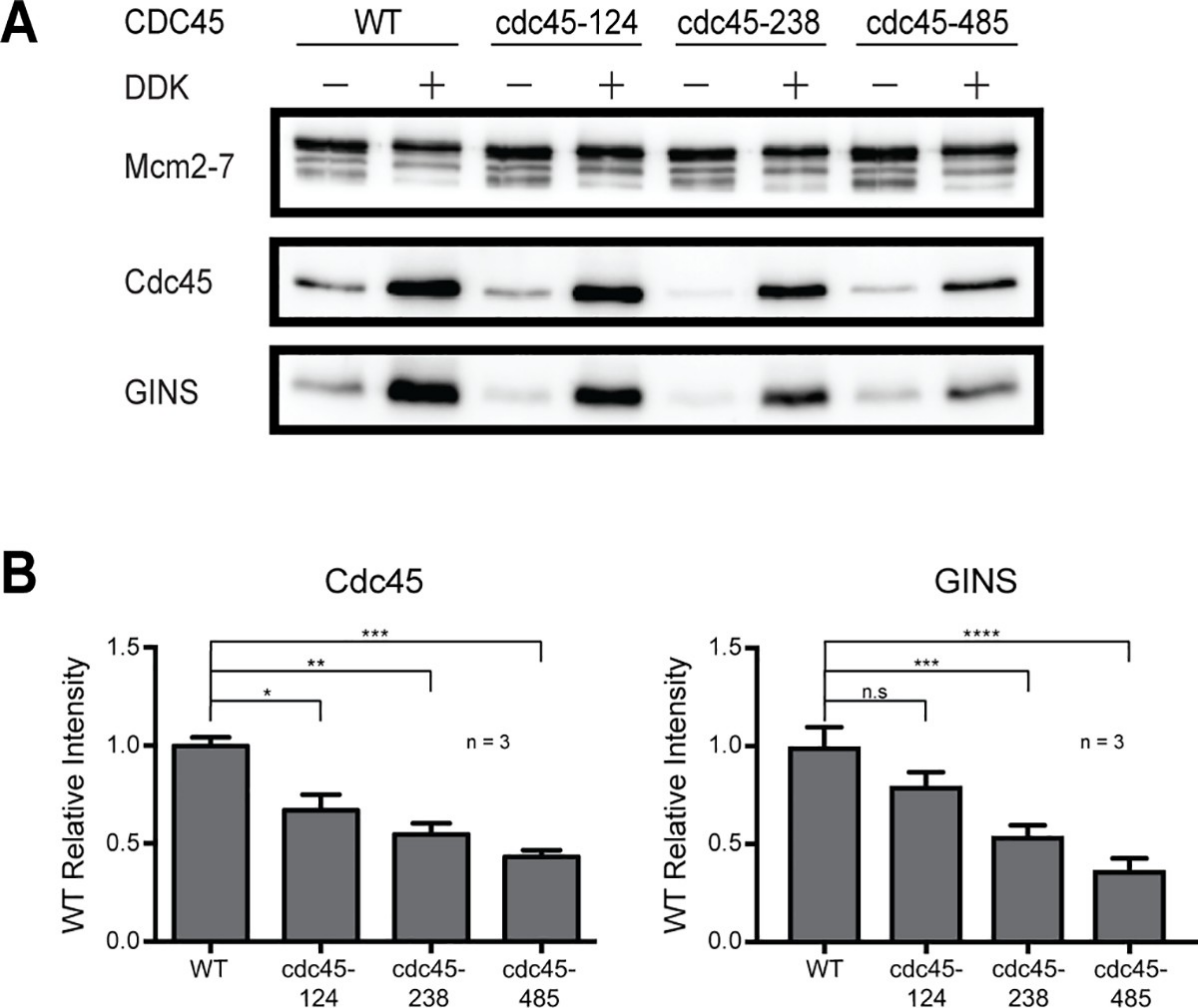
Cdc45-ts mutants are defective for CMG formation. (A) Proteins associated with the DNA at the end of the CMG formation assay. Bead-associated proteins were washed with Buffer H + 0.3M KCl, 0.02%NP-40, released with DNase and detected by immunoblot. (B) Relative association of Cdc45, Mcm2-7 and GINS with origin DNA after CMG-formation assay. Three experimental replicates were quantified and plotted. Error bars represent standard error from the mean. p≤0.01(**), p≤0.001(***), p≤0.0001(****), not significant (n.s., p≥0.05).

The previous *in vitro* experiments showed that Cdc45-124, Cdc45-238 and Cdc45-485 proteins showed defects in DNA association at the end of the replication reaction. However, the observed defects were in the context of reconstituted DNA replication reactions after CMG formation, activation and DNA synthesis. Thus, it is possible that mutant Cdc45-ts proteins are competent for initial CMG formation but became unstable during subsequent CMG activation or replication elongation. To investigate this possibility, we modified the DNA replication assay to allow CMG formation but prevented DNA synthesis by leaving out DNA Pol α/primase. Using this assay, we monitored salt-stable CMG complexes formed with each Cdc45-ts protein. As expected, wild-type Cdc45 was able to form stable CMG complexes on the DNA (Fig 6A, lane 1–2). The Cdc45-ts proteins showed similar but less dramatic defects during CMG formation as we observed in the replication assays (Fig 6A, lanes 3–8). The amount of Cdc45-124 stable CMG complexes was 67% of wild-type Cdc45 followed by Cdc45-238 (55%) and Cdc45-485 (43%) (Fig 6B). Consistent with these results, the amount of GINS associated as part of the mutant CMG complexes was reduced to similar levels (Fig 6B). Taken together, these findings show that the Cdc45-ts proteins are defective for initial CMG formation.

## Discussion

Our findings provide insights into the function of Cdc45 during DNA replication. We identified Cdc45 mutants selectively defective for DNA replication initiation but competent for DNA elongation *in vivo*. In addition, we demonstrated that Cdc45-124, Cdc45-238 and Cdc45-485 mutants are defective for DNA replication and CMG formation *in vitro*. Together, these data suggest that Cdc45 has different roles during DNA replication initiation and elongation. Alternatively, Cdc45 could have one function but replication initiation makes a more stringent demand on these requirements than does replication elongation. Our mutations also revealed essential roles for the RecJ-related and IDR regions of Cdc45, although the IDR as a whole is dispensable for Cdc45 function.

Structural and bioinformatic studies of Cdc45 demonstrate a clear homology between Cdc45 and RecJ [42–43]. We found two lethal and one temperature-sensitive mutations in the DHH-like domain of Cdc45 consistent with this domain being essential for Cdc45 function during DNA replication. Importantly, the Cdc45 RecJ temperature-sensitive mutation (*cdc45-124*) resulted in defects for DNA replication initiation and not elongation suggesting that the RecJ domain mediates at least one step during DNA replication initiation. During DNA replication initiation one DNA strand must be excluded from the central channel of the Mcm2-7 complex. Biochemical studies demonstrate that HsCdc45 free in solution has a weak affinity for DNA substrates [43–44]. It is possible that the DNA binding activity of Cdc45 assists this process by capturing the excluded DNA strand from the Mcm2-7 central channel. Conversely, it is possible the DHH-like domain of Cdc45 provides an essential structural scaffold for the rest of the Cdc45 protein. Consistent with the latter hypothesis, we were unable to purify the two lethal mutants in the DHH-like domain of Cdc45, although we were able to purify the Cdc45-124 protein.

Two other lethal mutants, *cdc45-171* and *cdc45-199*, are located in an intrinsic disordered region (IDR) within Cdc45. A similarly sized and located IDR is a common feature of Cdc45 proteins (S4 Fig), although these regions are not obviously related in sequence. Although these findings suggested that these disordered regions are important to Cdc45 function, we found that deletion of the *S*. *cerevisiae* IDR is dispensable for viability. Although this region is not visible in CMG structures, *in vitro* crosslinking studies suggest that it interacts with the catalytic subunit of DNA Pol ε (Pol2) [49]. Since Pol2 also interacts with Mcm2, Mcm5, Mcm6 and GINS [49], we propose that the IDR-Pol2 interactions are dispensable for Pol ε association with the CMG. Why then are mutations in the IDR lethal? One possibility is that *cdc45-171* and *cdc45-199* mutations cause conformational changes in the IDR that block the association of Pol ε with the CMG causing lethality. In addition, recent studies identified a region in the IDR required for Rad53 interaction with Cdc45 [47]. Disrupting the Cdc45-Rad53 interaction did not cause loss of viability in the presence of DNA damaging agents suggesting that this interaction is also dispensable for Rad53 function. These results support the idea that the IDR region of Cdc45 can serve as a scaffold for replication-fork-associated proteins. However, we cannot rule out the possibility that conformational changes in the IDR caused by *cdc45-171* and *cdc45-199* have a major effect in the local architecture of this region that can compromise the function of Cdc45 during DNA replication initiation or at replication forks.

The three Cdc45 temperature sensitive mutants we identified are defective for DNA replication initiation but not elongation. Each mutant arrests in G1-phase when shifted to the restrictive temperature and subsequently undergo reductional anaphase. This phenotype is also seen when Cdc45 is degraded in G1-arrested cells and allowed to enter S phase [34]. In contrast, arresting *cdc45-124*, *cdc45-238* and *cdc45-485* cells in G1-phase followed by incubation at the restrictive temperature and release retained cells with G1 DNA content without undergoing reductional anaphase. Because Cdc45 is recruited to early-firing origins in late G1-phase [50], it is possible that Cdc45-124, Cdc45-238 and Cdc45-485 molecules already associated with early origins during a G1 arrest are (partially or fully) protected from inactivation at the restrictive temperature. Once released at the restrictive temperature, these Cdc45 molecules could stimulate a low level of replication initiation that prevents reductional anaphase through checkpoint activation. Interestingly, the only other *CDC45* conditional allele, *cdc45-1*, is also primarily defective in initiation [51]. However, unlike the *CDC45* temperature-sensitive alleles described here, this allele has clear elongation defects including much slower progression through S phase after HU release [51].

Previous genetic studies have analyzed the effects of Cdc45 degradation at different stages of the cell cycle [34] but have not mapped the functional domains of Cdc45. We identified at least three regions that when mutated impair Cdc45 function. The Cdc45-124 mutation (DAH->AAA) alters Cdc45 residues that are located in the position of the RecJ DHH catalytic triad. Although Cdc45 has no nuclease activity, the integrity of this domain may be structurally relevant for Cdc45 function during DNA replication initiation. The residues altered in Cdc45-238 (EEY->AAA) are located at the end of an α-helix adjacent to the IDR domain in Cdc45. The IDR domain is not visible in the structure and it is thought to interact with Pol ε [49]. Since Pol ε association is required for CMG formation and activation [52], it is possible that Cdc45-238 initiation defects are due to defective Pol ε association with the IDR. Finally, the residues altered in Cdc45-485 (DDRK->AAAA) are located at the surface of Cdc45 facing GINS. In the available CMG structures [20, 23, 26], this region is in close proximity to the Psf3 subunit of GINS without any specific protein-protein interactions. It remains possible, however, that interaction between these regions are required during CMG formation or helicase activation.

There are at least two possible steps that could be defective in Cdc45-124, Cdc45-238 and Cdc45-485 mutants. One possibility is that they are defective for initial CMG formation with loaded Mcm2-7 helicases. Second, it is possible that they can form a CMG but the resulting complex becomes unstable upon helicase activation. This second possibility is less likely because such an effect would most likely impact elongation. Consistent with the former hypothesis, all three mutants are defective for CMG formation (Fig 6).

Our findings suggest that Cdc45 function during helicase activation is different than its role during DNA elongation. There are two possible explanations for the separation of function phenotypes that we observe for the temperature-sensitive mutations we identified. One possibility is that Cdc45 performs at least one distinct function during replication initiation that is not required during replication elongation. In this case, even though they are located in different parts of the protein each of the Cdc45-ts alleles described here would have targeted a region of Cdc45 involved in this function. Alternatively, it is possible that the Cdc45-ts proteins can only be inactivated if they are free in solution. This type of defect could explain the lack of elongation defects since the mutant Cdc45 proteins would have already been incorporated into CMG complexes at replication forks in the experiments testing for elongation. In this case, Cdc45 interactions with GINS and Pol ε could protect Cdc45-ts proteins from inactivation. Alternatively, it is possible that Cdc45 might undergo a conformational change upon CMG activation that prevent Cdc45-ts inactivation in the context of the CMG complex.

Although we did not identify mutants that were specifically defective in DNA replication elongation, we cannot rule out the possibility that such mutants could be found. Degradation of Cdc45 during DNA elongation stops fork movement [34]. Since Cdc45 stimulates Mcm2-7 ATPase activity [15], it is possible that Cdc45 mutants defective in Mcm2-7 ATPase stimulation but not CMG formation would be defective for DNA replication elongation. Alternatively, if all the Cdc45 functions required for elongation are also required for helicase activation (e.g. Mcm2-7 ATPase activity has recently been proposed to be required for helicase activation [18]), identifying elongation-specific Cdc45 mutations may not be possible.

## Supporting information

S1 Fig *CDC45* sequence alignment. *CDC45* sequence alignment of indicated species was obtained using EMBL-EBI Clustal Omega. The Intrinsic Disordered Region (IDR) and nuclear localization signal (NLS) of *S*. *cerevisiae* are underlined in red and purple, respectively. Site-directed mutants are underlined in black and labeled by the position of the first mutated amino acid. Blue and yellow bars indicate very similar and identical residues, respectively. Secondary structures were adapted from Yuan, Z., et al. (2016). Red spirals and gray arrows denote α-helices and β-strands, respectively. The α-helices and β-strands belonging to the DDH domain from RecJ were adapted from Simon, A.C., et al (2016).
(PDF)

S2 Fig *CDC45* mutants are not sensitive to hydroxyurea (HU) and methyl methanesulfonate (MMS). Five-fold serial dilutions of viable *cdc45* mutants were grown on indicated plates for 4d at 25°C.
(TIF)

S3 Fig Structural diagram of Cdc45 temperature-sensitive and lethal mutants. (A) Structure of Cdc45 (shown in gray) in the context of the CMG from Saccharomyces cerevisiae. Mcm2, Mcm5 and GINS subunits are differentially colored and labeled. Mcm3, Mcm4, Mcm6 and Mcm7 were omitted for clarity. The IDR region of Cdc45 is shown as dashed lines. (B) Isolated structure of Cdc45 in the context of the CMG as shown in (A). Left Location of Cdc45 temperature-sensitive mutants are shown in red. Right Location of Cdc45 lethal mutants are shown in red. Cdc45-171 and Cdc45-199 lethal mutants not visible in the structure are shown as part of the IDR (dashed lines). CMG and Cdc45 structures were adapted from Yuan, Z., et al (2016).
(TIF)

S4 Fig The DHH-motif mutant of *CDC45* is viable. A mutation in *CDC45* that restores the catalytic-triad involved in RecJ ssDNA nuclease activity is viable. Four clones of *cdc45-DAH124DHH* mutant cells were streaked on 5-FOA plates and grown for 2d at 25°C.
(TIF)

S5 Fig The presence of an intrinsic disordered region in Cdc45 is conserved. PONDR protein disorder prediction was used to analyze indicated Cdc45 protein (Sc = *S*. *cerevisiae*, Sp = *S*. *pombe*, Dm = *D*. *melanogaster*, Xl = *X*. *laevis*, Hs = *H*. *sapiens*). Highest confidence protein regions predicted to be disordered are shown as dotted lines (Sc = 169–229, Sp = 153–215, Dm = 125–196, Xl = 129–183, Hs = 128–182). In each case, this region is found after the RecJ homology region of Cdc45.
(TIF)

S6 Fig The *cdc45-ΔIDR* mutant is not sensitive to hydroxyurea. Cells whose only copy of the CDC45 gene was deleted for the Cdc45 intrinsic-disordered region (IDR) were tested for sensitivity to hydroxyurea (HU). Five-fold serial dilutions of viable *cdc45* mutants were grown on the indicated plates for 4d at 25°C.
(TIF)

S1 Table Yeast strains used in this study. (PDF)

S2 Table Plasmid DNAs used in this study. (PDF)
