# Supplementary figures and images for: Initiation-specific alleles of the Cdc45 helicase-activating protein
Source: PLoS One. 2019 Mar 26;14(3):e0214426. doi: 10.1371/journal.pone.0214426 (PMC6435160; doi:10.1371/journal.pone.0214426)

S1 Fig. CDC45 sequence alignment.

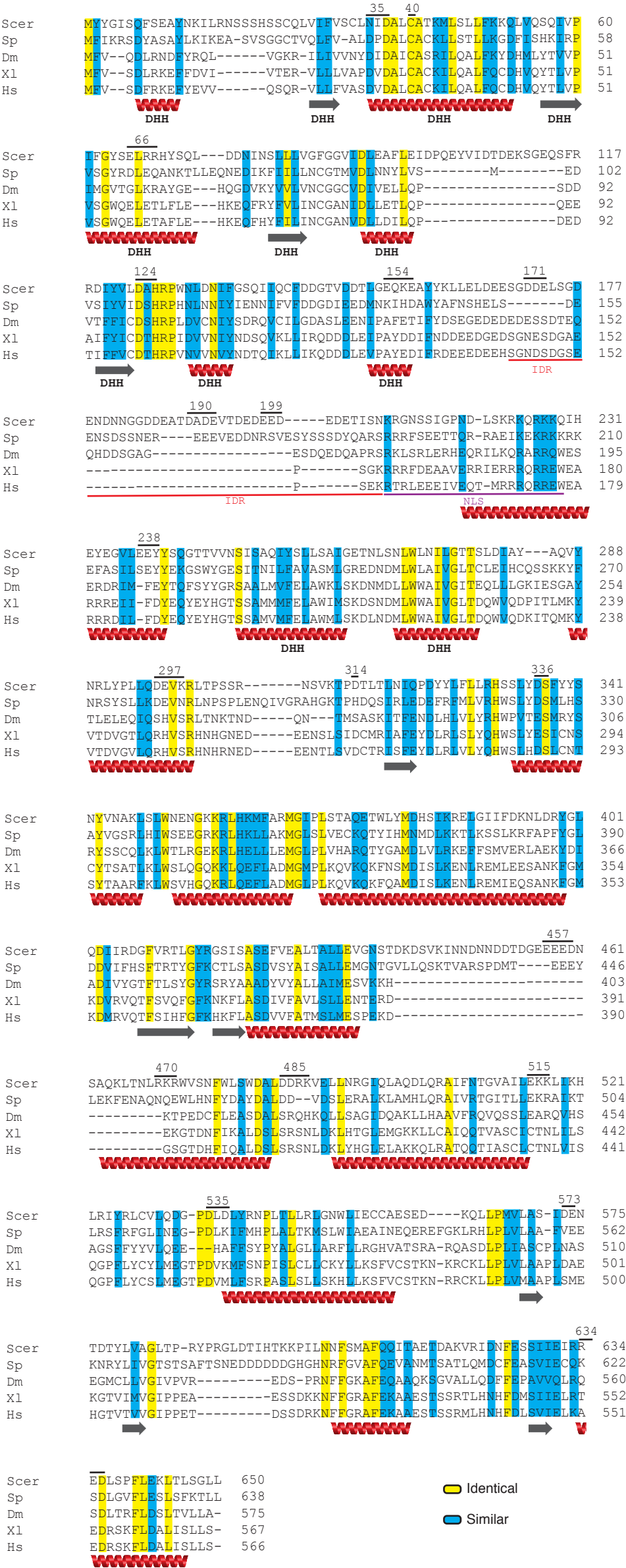

Supplement: S1 Fig — CDC45 sequence alignment of indicated species was obtained using EMBL-EBI Clustal Omega. The Intrinsic Disordered Region (IDR) and nuclear localization signal (NLS) of S. cerevisiae are underlined in red and purple, respectively. Site-directed mutants are underlined in black and labeled by the position of the first mutated amino acid. Blue and yellow bars indicate very similar and identical residues, respectively. Secondary structures were adapted from Yuan, Z., et al. (2016). Red spirals and gray arrows denote α-helices and β-strands, respectively. The α-helices and β-strands belonging to the DDH domain from RecJ were adapted from Simon, A.C., et al (2016). (PDF) [file pone.0214426.s001.pdf]

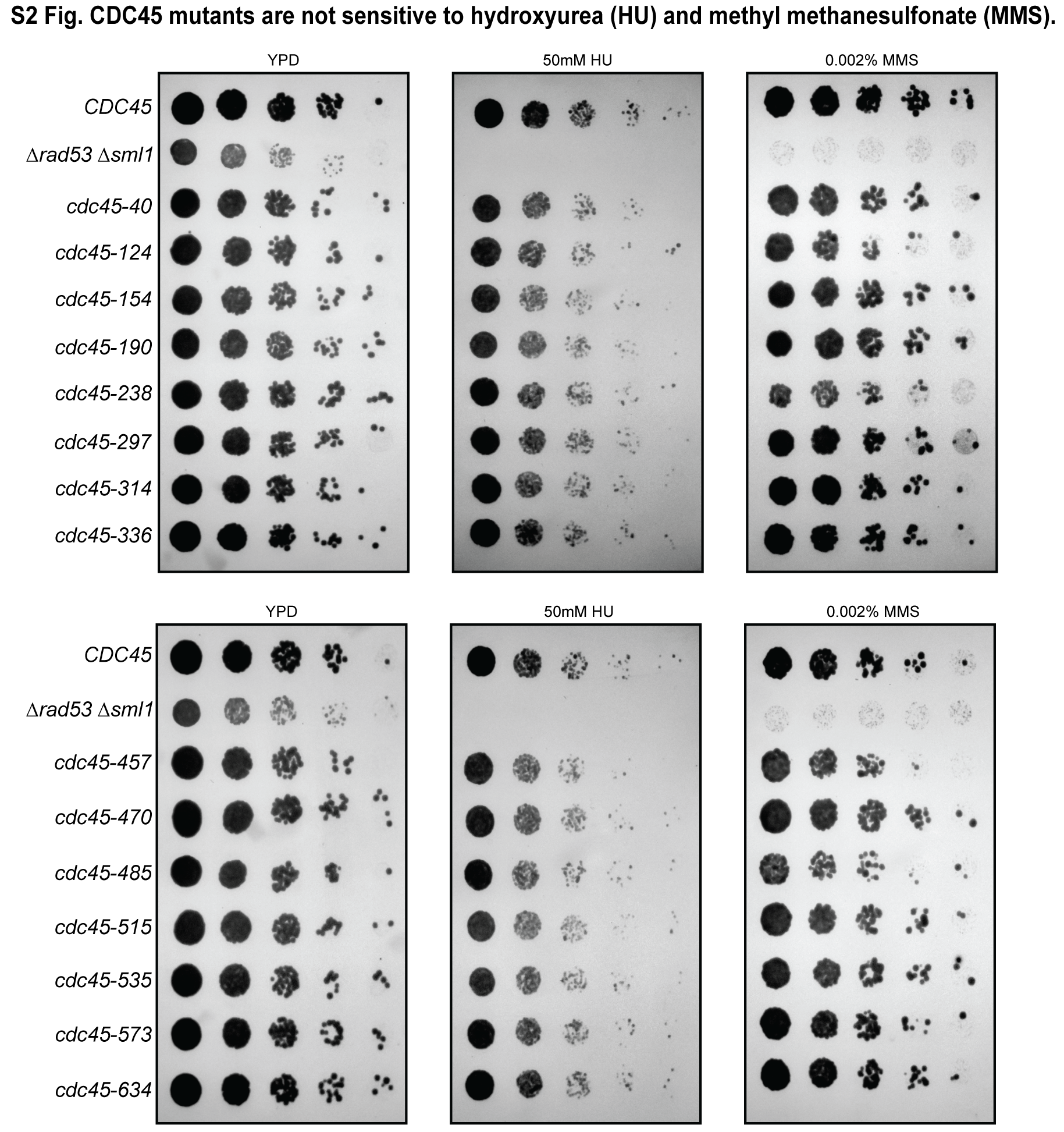

Supplement: S2 Fig — Five-fold serial dilutions of viable cdc45 mutants were grown on indicated plates for 4d at 25°C. (TIF) [file pone.0214426.s002.tif]

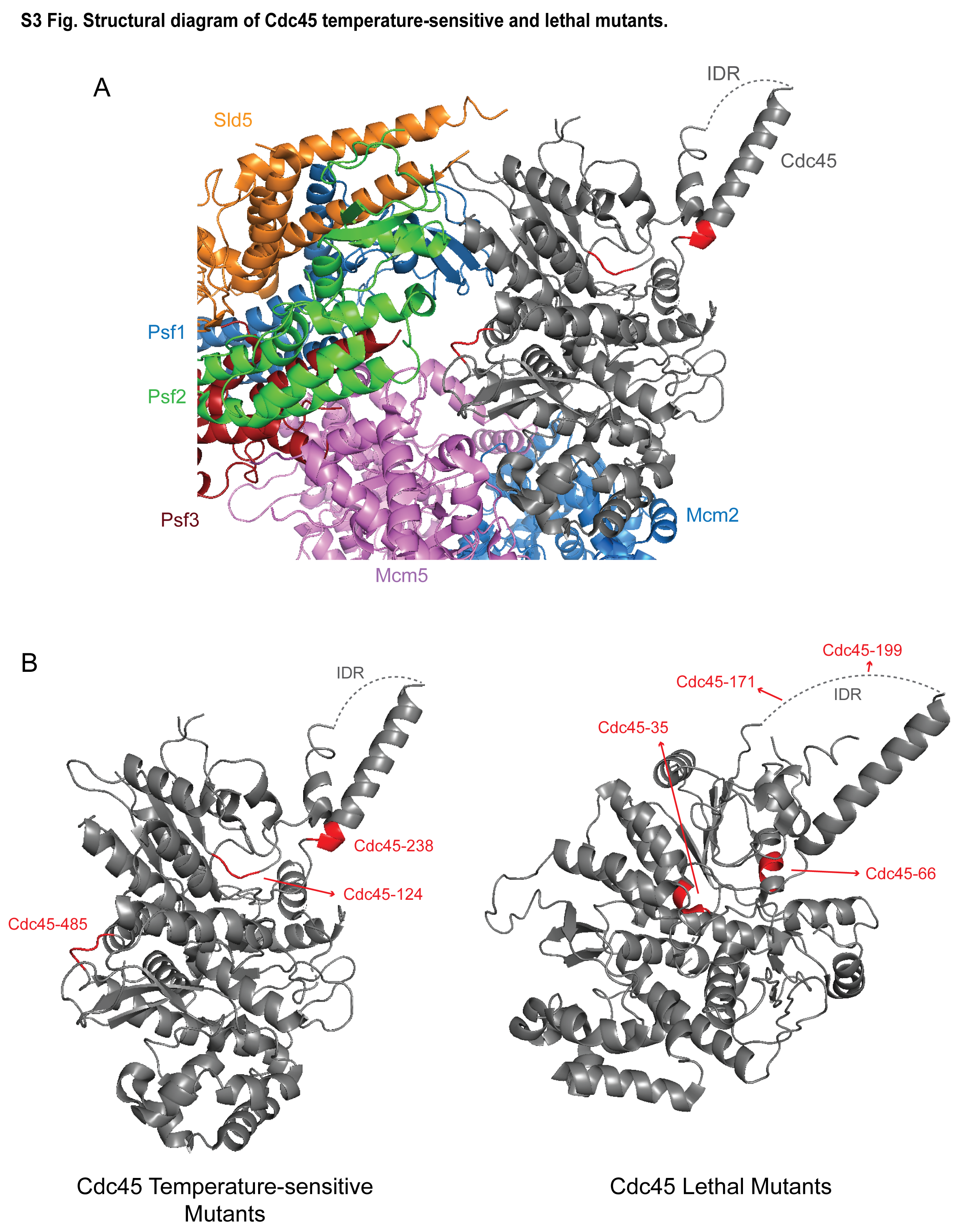

Supplement: S3 Fig — (A) Structure of Cdc45 (shown in gray) in the context of the CMG from Saccharomyces cerevisiae. Mcm2, Mcm5 and GINS subunits are differentially colored and labeled. Mcm3, Mcm4, Mcm6 and Mcm7 were omitted for clarity. The IDR region of Cdc45 is shown as dashed lines. (B) Isolated structure of Cdc45 in the context of the CMG as shown in (A). Left Location of Cdc45 temperature-sensitive mutants are shown in red. Right Location of Cdc45 lethal mutants are shown in red. Cdc45-171 and Cdc45-199 lethal mutants not visible in the structure are shown as part of the IDR (dashed lines). CMG and Cdc45 structures were adapted from Yuan, Z., et al (2016). (TIF) [file pone.0214426.s003.tif]

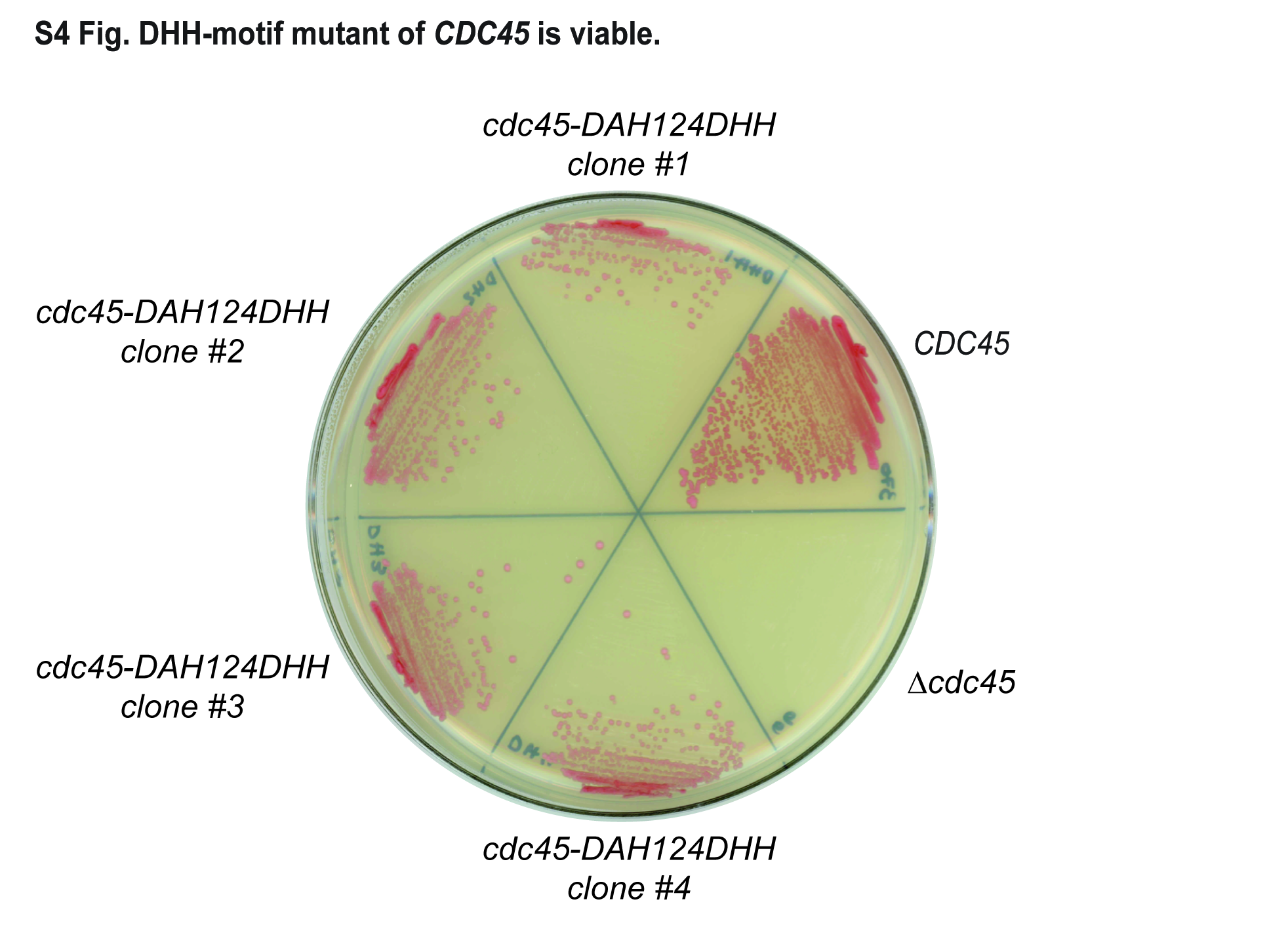

Supplement: S4 Fig — A mutation in CDC45 that restores the catalytic-triad involved in RecJ ssDNA nuclease activity is viable. Four clones of cdc45-DAH124DHH mutant cells were streaked on 5-FOA plates and grown for 2d at 25°C. (TIF) [file pone.0214426.s004.tif]

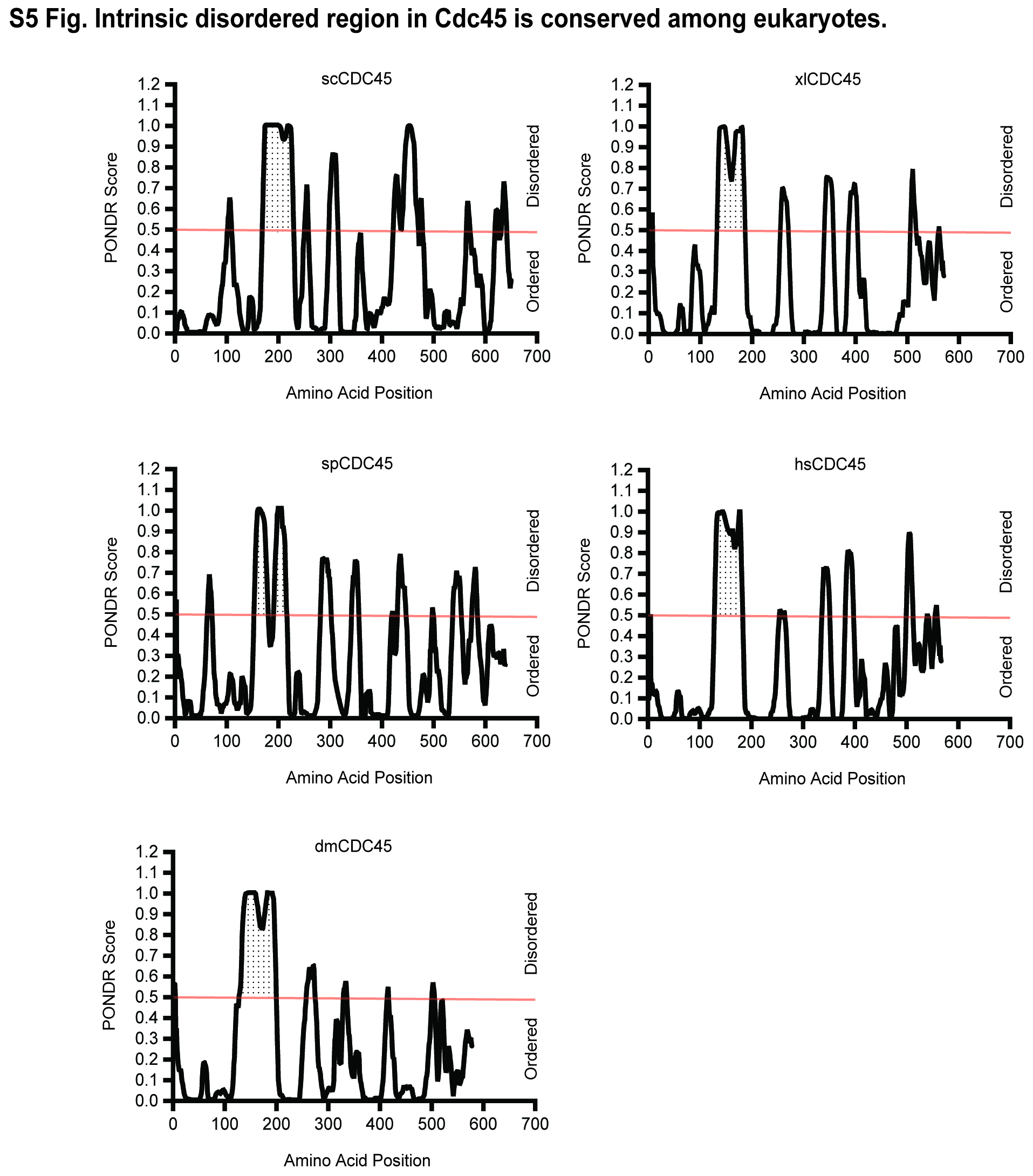

Supplement: S5 Fig — PONDR protein disorder prediction was used to analyze indicated Cdc45 protein (Sc = S. cerevisiae, Sp = S. pombe, Dm = D. melanogaster, Xl = X. laevis, Hs = H. sapiens). Highest confidence protein regions predicted to be disordered are shown as dotted lines (Sc = 169–229, Sp = 153–215, Dm = 125–196, Xl = 129–183, Hs = 128–182). In each case, this region is found after the RecJ homology region of Cdc45. (TIF) [file pone.0214426.s005.tif]

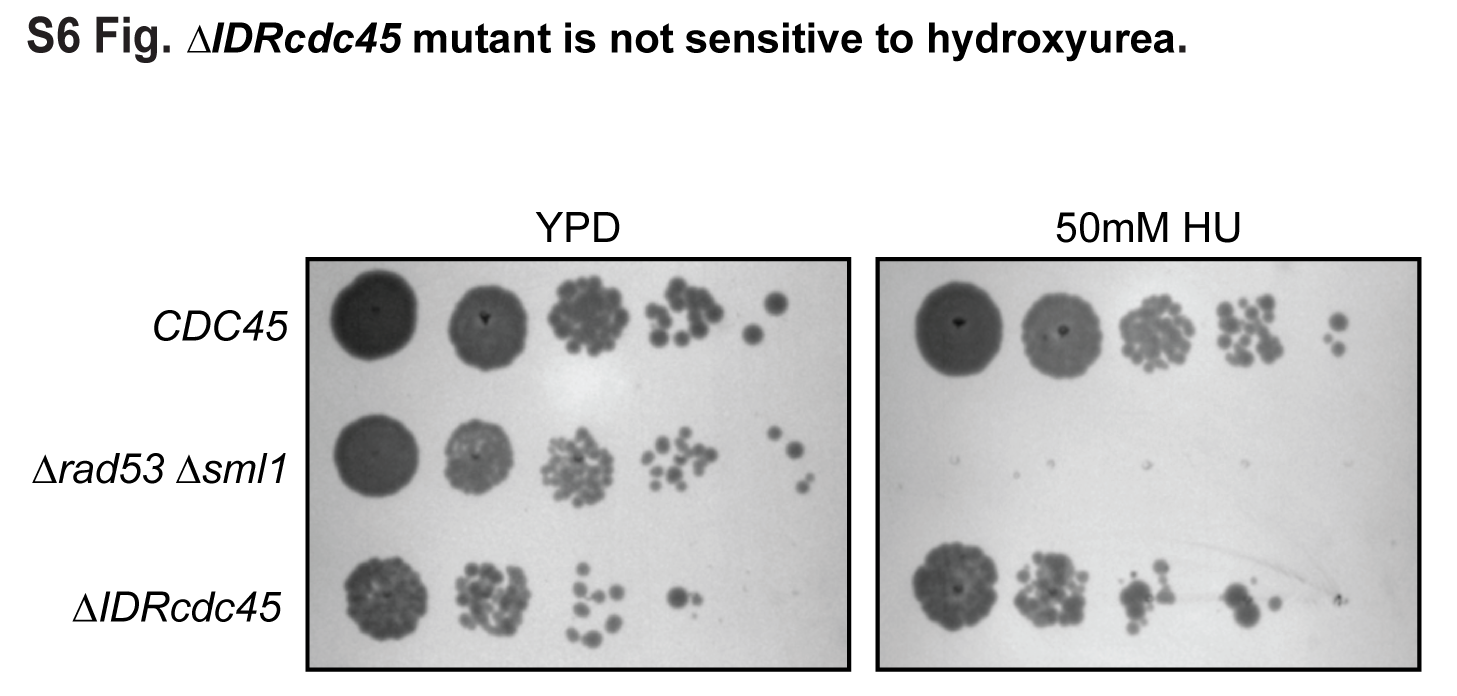

Supplement: S6 Fig — Cells whose only copy of the CDC45 gene was deleted for the Cdc45 intrinsic-disordered region (IDR) were tested for sensitivity to hydroxyurea (HU). Five-fold serial dilutions of viable cdc45 mutants were grown on the indicated plates for 4d at 25°C. (TIF) [file pone.0214426.s006.tif]
