# Supplementary material for: Initiation-specific alleles of the Cdc45 helicase-activating protein
Source: PLoS One. 2019 Mar 26;14(3):e0214426. doi: 10.1371/journal.pone.0214426 (PMC6435160; doi:10.1371/journal.pone.0214426)
Supplement: S1 Table — (PDF) [file pone.0214426.s007.pdf]

S1 Table. Yeast strains used in this study.

Strain Name	Genotype	Purpose	Source
yBC093	MATα cdc45::KanMX pRS416-CDC45	Cdc45 plasmid shuffle	This study
yRR01	MATa cdc45::cdc45-124 bar1::TRP1	Cdc45 plasmid shuffle	This study
yRR02	MATa cdc45::cdc45-238 bar1::TRP1	Cdc45 plasmid shuffle	This study
yRR03	MATa cdc45::KanMX LEU2::pRR01-cdc45-485	Cdc45 plasmid shuffle	This study
yRR04	MATα cdc45::KanMX LEU2::pRR01-cdc45-535	Cdc45 plasmid shuffle	This study
yRR05	MATα cdc45::KanMX LEU2::pRR01-cdc45-336	Cdc45 plasmid shuffle	This study
yRR06	MATα cdc45::KanMX LEU2::pRR01-cdc45-470	Cdc45 plasmid shuffle	This study
yRR07	MATα cdc45::KanMX LEU2::pRR01-cdc45-515	Cdc45 plasmid shuffle	This study
yRR08	MATα cdc45::KanMX LEU2::pRR01-cdc45-573	Cdc45 plasmid shuffle	This study
yRR09	MATα cdc45::KanMX LEU2::pRR01-cdc45-634	Cdc45 plasmid shuffle	This study
yBC49	MATα cdc45::KanMX LEU2::pRR01-cdc45-35 pRS416-CDC45	Cdc45 plasmid shuffle	This study
yBC50	MATα cdc45::KanMX LEU2::pRR01-cdc45-66 pRS416-CDC45	Cdc45 plasmid shuffle	This study
yBC51	MATα cdc45::KanMX LEU2::pRR01-cdc45-40 pRS416-CDC45	Cdc45 plasmid shuffle	This study
yBC53	MATα cdc45::KanMX LEU2::pRR01-cdc45-154 pRS416-CDC45	Cdc45 plasmid shuffle	This study
yBC55	MATα cdc45::KanMX LEU2::pRR01-cdc45-190 pRS416-CDC45	Cdc45 plasmid shuffle	This study
yBC58	MATα cdc45::KanMX LEU2::pRR01-cdc45-297 pRS416-CDC45	Cdc45 plasmid shuffle	This study
yBC59	MATα cdc45::KanMX LEU2::pRR01-cdc45-314 pRS416-CDC45	Cdc45 plasmid shuffle	This study
yBC61	MATα cdc45::KanMX LEU2::pRR01-cdc45-457 pRS416-CDC45	Cdc45 plasmid shuffle	This study
yRR10	MATα cdc45::KanMX LEU2::pRR01-cdc45-171 pRS416-CDC45	Cdc45 plasmid shuffle	This study
yRR11	MATα cdc45::KanMX LEU2::pRR01-cdc45-199 pRS416-CDC45	Cdc45 plasmid shuffle	This study
yRR12	MATα cdc45::KanMX LEU2::pRR01-cdc45Δ169-209	Cdc45 plasmid shuffle	This study
yRR23	MATa pep4::unmarked LEU2::GAL-cdc45-124 3xFlag	Cdc45 purification	This study
yRR24	MATa pep4::unmarked LEU2::GAL-cdc45-238 3xFlag	Cdc45 purification	This study
yRR25	MATa pep4::unmarked LEU2::GAL-cdc45-485 3xFlag	Cdc45 purification	This study
yMM16	MATa pep4::unmarked LEU2::GAL-CDC45 3xFlag	Cdc45 purification	(17)
ySK136	MATa pep4::unmarked bar1::hisG URA3::pGAL-SLD5 LEU2::pGAL-PSF2-3C-his-Flag LYS2::pGAL-PSF1+PSF3	GINS purification	(17)
ySK123	MATa pep4::unmarked bar1::hisG LEU2::pGAL1-Δ1-104-SLD3-3xFlag HIS3::pGAL-SLD7-VSV-G	Sld3/7 purification	(17)
ySK127	MATa pep4::unmarked bar1::hisG LEU2::pGAL-3xFlag-3C-SLD2	Sld2 purification	(17)
