# Supplementary material for: Initiation-specific alleles of the Cdc45 helicase-activating protein
Source: PLoS One. 2019 Mar 26;14(3):e0214426. doi: 10.1371/journal.pone.0214426 (PMC6435160; doi:10.1371/journal.pone.0214426)
Supplement: S2 Table — (PDF) [file pone.0214426.s008.pdf]

S2 Table. DNA vectors used in this study.

Plasmid Name	Description	Purpose	Source
pRR01	pRS405-*CDC45*	Cdc45 plasmid shuffle	This study
pRR02	pRS405-*cdc45-171*	Cdc45 plasmid shuffle	This study
pRR03	pRS405-*cdc45-199*	Cdc45 plasmid shuffle	This study
pRR04	pRS405-*cdc45Δ169-209*	Cdc45 plasmid shuffle	This study
pRR05	pRS405-*cdc45-124*	Cdc45 plasmid shuffle	This study
pRR06	pRS405-*cdc45-238*	Cdc45 plasmid shuffle	This study
pRR07	pRS405-*cdc45-485*	Cdc45 plasmid shuffle	This study
pRR08	pRS405-*Gal1,10 cdc45-124-3x-Flag*	Cdc45 purification	This study
pRR09	pRS405-*Gal1,10 cdc45-238-3x-Flag*	Cdc45 purification	This study
pRR10	pRS405-*Gal1,10 cdc45-485-3x-Flag*	Cdc45 purification	This study
pBC35	pRS405-*cdc45-35-3x-HA-3x-Flag*	Cdc45 plasmid shuffle	This study
pBC36	pRS405-*cdc45-66-3x-HA-3x-Flag*	Cdc45 plasmid shuffle	This study
pBC38	pRS405-*cdc45-40-3x-HA-3x-Flag*	Cdc45 plasmid shuffle	This study
pBC39	pRS405-*cdc45-154-3x-HA-3x-Flag*	Cdc45 plasmid shuffle	This study
pBC41	pRS405-*cdc45-190-3x-HA-3x-Flag*	Cdc45 plasmid shuffle	This study
pBC44	pRS405-*cdc45-297-3x-HA-3x-Flag*	Cdc45 plasmid shuffle	This study
pBC45	pRS405-*cdc45-314-3x-HA-3x-Flag*	Cdc45 plasmid shuffle	This study
pBC46	pRS405-*cdc45-336-3x-HA-3x-Flag*	Cdc45 plasmid shuffle	This study
pBC47	pRS405-*cdc45-457-3x-HA-3x-Flag*	Cdc45 plasmid shuffle	This study
pBC48	pRS405-*cdc45-470-3x-HA-3x-Flag*	Cdc45 plasmid shuffle	This study
pRR11	pRS405-*cdc45-515*	Cdc45 plasmid shuffle	This study
pBC54	pRS405-*cdc45-535-3x-HA-3x-Flag*	Cdc45 plasmid shuffle	This study
pRR12	pRS405-*cdc45-573*	Cdc45 plasmid shuffle	This study
pBC56	pRS405-*cdc45-634-3x-HA-3x-Flag*	Cdc45 plasmid shuffle	This study
pMM33	pRS405-*Gal1,10 CDC45-3x-Flag*	Cdc45 purification	(17)
